# Comparison of Publication of Pediatric Probiotic vs Antibiotic Trials Registered on ClinicalTrials.gov

**DOI:** 10.1001/jamanetworkopen.2021.25236

**Published:** 2021-10-08

**Authors:** Madison Riddell, Kaden Lam, Anna Funk, Nidhi Lodha, Diane L. Lorenzetti, Stephen B. Freedman

**Affiliations:** 1 Department of Pediatrics, Alberta Children’s Hospital, Cumming School of Medicine, University of Calgary, Calgary, Alberta, Canada; 2 Section of Pediatric Emergency Medicine, Department of Pediatrics, Alberta Children’s Hospital, Cumming School of Medicine, University of Calgary, Calgary, Alberta, Canada; 3 Department of Community Health Sciences and the Health Sciences Library, University of Calgary, Calgary, Alberta, Canada; 4 Sections of Pediatric Emergency Medicine and Gastroenterology, Department of Pediatrics, Alberta Children’s Hospital Research Institute, Cumming School of Medicine, University of Calgary, Calgary, Alberta, Canada

## Abstract

**Importance:**

The published evidence in support of probiotic use is conflicting, which may be a result of selective publication of probiotic trials.

**Objectives:**

To compare the proportion of registered trials that evaluate pediatric probiotics vs those that evaluate antibiotics that are published and to identify study-related factors associated with publication status.

**Design, Setting, and Participants:**

This cross-sectional study evaluated eligible trials registered in ClinicalTrials.gov, an online clinical trials registry, from July 1, 2005, to June 30, 2016. Eligible studies included participants younger than 18 years, evaluated a probiotic or 1 of the 5 most commonly prescribed antibiotics in children and adolescents, and randomized study participants. All searches were updated and finalized as of September 9, 2020.

**Exposures:**

Probiotic or antibiotic.

**Main Outcomes and Measures:**

The primary outcome was study publication status. In addition, exposure status (probiotic vs antibiotic), trial result, and funding source were assessed for independent association with publication status. Whether study design elements, publication journal impact factor, and the interval from study completion to publication differed by exposure status were also evaluated.

**Results:**

A total of 401 unique trials (265 probiotic and 136 antibiotic) met eligibility criteria. A greater proportion of antibiotic compared with probiotic studies were published (83 [61.0%] vs 119 [44.9%]; difference, 16.1% [95% CI, 5.8%-25.9%]). After adjustment for funding source, blinding, and purpose, studies evaluating an antibiotic were more likely to be published (odds ratio, 2.1 [95% CI, 1.3-3.4]). No other covariates included in the model were independently associated with publication status. Antibiotic trials, compared with probiotic trials, were more likely to have a therapeutic purpose (114 [83.8%] vs 117 [44.2%]; difference, 39.6% [95% CI, 31.1%-48.3%]) and to be multicenter (46 [33.8%] vs 46 [17.4%]; difference, 16.5% [95% CI, 7.5%-25.7%]). The median impact factor of the journals in which the studies were published was higher for the antibiotic trials (7.2 [IQR, 2.8-20.5] vs 3.0 [IQR, 2.3-4.2]; *P* < .001). The median number of days to publication did not differ between the probiotic and antibiotic trials (683 [IQR, 441-1036] vs 801 [IQR, 550-1183]; *P* = .24).

**Conclusions and Relevance:**

The findings of this cross-sectional study suggest that probiotic studies are less likely to be published than antibiotic trials. No other study characteristics were associated with publication status. This finding raises concerns regarding the results of meta-analyses of probiotic trials.

## Key Points

**Question:**

Do registered trials that evaluate the use of probiotics and routinely used antibiotics in children differ in their publication rates?

**Findings:**

This cross-sectional analysis of 265 unique probiotic trials and 136 unique antibiotic trials in the ClinicalTrials.gov registry found that among registered trials conducted in children, probiotic trials are less likely to be published than antibiotic trials after adjustment for funding source, presence of blinding, and study purpose. No other evaluated study characteristics were independently associated with publication status in the regression model.

**Meaning:**

These findings suggest that pediatric probiotic trials may be more prone to selective publication compared with pediatric antibiotic trials; therefore, meta-analyses and systematic reviews of probiotics must be interpreted cautiously.

## Teaser

This cross-sectional study compares the proportion of published registered trials that evaluate the use of pediatric probiotics vs those that evaluate the use of routinely used pediatric antibiotics.

## Introduction

Probiotics represent a rapidly growing commercial industry valued at approximately US $15 billion in 2013.^1^ With growth estimated to be 7% per annum,^2^ there has been a concerted effort to document clinical effectiveness^1,3,4^ through the conduct of randomized clinical trials that are summarized in numerous meta-analyses.^5,6,7,8,9,10^ The latter inform guideline recommendations,^11,12^ which often support probiotic use with the caveat that the evidence is weak owing to the inclusion of studies with a high risk of bias.^13,14,15^ Although many early studies that influence the conclusions of meta-analyses reported benefits associated with probiotic use, recent multicenter trials often contradict these results,^2,4,9,16,17^ and they are beginning to inform the conclusions of reviews. For example, the 2010 Cochrane review of probiotic use in acute infectious diarrhea (63 studies) concluded that probiotics “have clear beneficial effects in shortening the duration and reducing stool frequency in acute infectious diarrhoea.”^18^^(p2)^ However, the 2020 update (82 studies) states that “probiotics probably make little or no difference to the number of people who have diarrhoea lasting 48 hours or longer, and we are uncertain whether probiotics reduce the duration of diarrhoea.”^19^^(p2)^

Publication bias occurs when the direction or strength of a finding influences the likelihood, timing, language, and journal of publication.^20^ This phenomenon may explain the discordant conclusions of probiotic meta-analyses and the results of recent large, multicenter clinical trials.^21^ Because 25% to 50% of registered trials remain unpublished after study completion, meta-analyses may reach incorrect conclusions if the included data misrepresent the effects, benefits, and risks of the intervention.^22,23^ Publication bias is a particular concern in fields where industry-funded studies are common because they are associated with study discontinuation, nonpublication, and more favorable reporting of results and conclusions.^24,25^

The International Committee of Medical Journal Editors has embraced the use of registries, such as ClinicalTrials.gov, and since 2005 have required trial registration before participant enrollment as a prerequisite for publication.^26^ We therefore used this data source to compare the proportion of registered trials that are published between those evaluating a probiotic relative to those evaluating an antibiotic. Our secondary objective was to determine whether exposure status (ie, probiotic or antibiotic), trial result, or funding source were independently associated with publication status and whether study design elements, journal impact factor, and interval from study completion to publication differed by exposure status. We hypothesized that a lower proportion of registered probiotic trials are published compared with antibiotic trials.

## Methods

### Study Design and Setting

This cross-sectional study used publicly available aggregate data; thus, institutional review board approval was not required. The study followed the Strengthening the Reporting of Observational Studies in Epidemiology (STROBE) reporting guideline.

The study included trials registered in ClinicalTrials.gov with start dates from July 1, 2005, to June 30, 2016. Established in 1999, ClinicalTrials.gov is an online, publicly available registry that includes details on interventions, outcomes, results, and funding sources. The start date was selected to correspond with the 2005 International Committee of Medical Journal Editors requirement that trials be registered in advance of publication.^27^ The end date was chosen to allow for a sufficient interval between registration and study publication. Eligible studies met the following criteria: (1) the included participants were younger than 18 years (identified as children in the database); (2) the participants were randomized to at least 2 alternate interventions; and (3) the study evaluated a probiotic or 1 of the 5 most commonly prescribed antibiotics in children (eTable 1 in the Supplement).^28,29^ Antibiotics were selected to serve as the referent standard because these medications are no longer patent protected, and the financial implications of trial results would be minimal for industry stakeholders.

The registry search (eTable 2 in the Supplement) was undertaken with the assistance of a medical librarian (D.L.L.) using search terms for probiotics (*Lactobacillus* or *probiotic* or *Saccharomyces* or *Enterococcus* or *Streptococcus* or *Acidophilus*) and antibiotics (*azithromycin* or *amoxicillin–clavulanic acid* or *amoxicillin* or *cefdinir* or *cephalexin*). These terms were exploded to include synonyms through the ClinicalTrials.gov advanced search function (eTables 1 and 3 in the Supplement). Our search was limited to interventional trials. We did not apply any study purpose, language, result, or recruitment status restrictions. After the initial search, data were downloaded from ClinicalTrials.gov via comma-separated values export for review.

### Eligibility Screening

All searches were updated and finalized as of September 14, 2020. Two authors (M.R. and K.L.) independently performed the search and evaluated studies for eligibility. After completion of independent screening and removal of duplicates, disagreements were resolved via consultation with a third reviewer (S.B.F.).

### Data Extraction and Definitions

Two reviewers from a group of 3 (M.R., K.L., or N.L.) extracted data independently into a standardized database that was piloted with the first 10 eligible studies and subsequently refined before completing extraction for the remaining studies. If discrepancies occurred, the 2 reviewers attempted to resolve them; if discrepancies remained, a third reviewer (S.B.F.) was consulted.

Study completion was the date when participants were no longer being examined or treated (ie, final visit had occurred), as stated in the ClinicalTrials.gov registry. When this data field was incomplete, the estimated date of completion was extracted. Study duration was the interval from study start to completion dates. Data extractors classified the study purpose based on the following predefined criteria: therapeutic studies used an investigational agent to improve a specified medical condition; prophylactic studies evaluated investigational agent use for preventative purposes; safety/tolerability studies determined adverse effects associated with the investigational agent; and basic science studies were designed to assess pharmacokinetic properties. Data extractors classified the comparison group as placebo, standard care if the study described comparison to standard of care treatment, separate intervention if the comparison was another therapeutic agent, or alternate dosing if the trial compared alternative methods of administration, timing, or dosing of the intervention.

Blinding was present if any level of masking was reported on ClinicalTrials.gov. Trials are categorized by the registry into the following population age groups: premature infants (gestational age <36 weeks), term infants (aged 0-15 months), all ages (15 months to 18 years), and children and adults (includes participants older than 18 years). We categorized funding source according to the study sponsor disclosed on ClinicalTrials.gov. If any industry funding was disclosed, the study was categorized as industry funded.

Published trials had the following additional data extracted: whether findings for all primary outcomes were reported, direction of results, date, and journal of publication. We classified a study finding as positive if the results of the primary outcome, as stated in ClinicalTrials.gov, were statistically significant (ie, based on reported *P* values and/or 95% CIs).^30^ For noninferiority trials, if the investigational agent was found to be noninferior, the results were classified as positive. If there were multiple primary outcomes and the results were not uniformly positive or negative, these studies were classified as mixed. The publication interval was the difference between the study completion date (actual or estimated) and the date of publication. Journal impact factor was determined using the 2019 edition of Journal Citation Reports (Clarivate Analytics).^31^

### Outcomes

#### Primary Outcome

Publication status, the primary outcome, was assessed in accordance with previous work in the field.^23^ First, the ClinicalTrials.gov registry was examined to identify whether an article citation was provided. If a citation was not identified, a PubMed search was performed using the ClinicalTrials.gov NCT number. If a publication was not located, PubMed was subsequently searched using the name of the principal investigator and other identifying descriptors (eg, population, intervention, and study outcomes).^30^ If a matching publication was not identified, the strategy was repeated using Google Scholar. All searches were updated and finalized as of September 9, 2020.

#### Secondary Objectives

We sought to determine whether study intervention (ie, probiotic vs antibiotic), outcome (ie, positive vs negative vs mixed), or funding source (ie, industry vs nonindustry) were independently associated with publication status. In addition, we aimed to determine whether study design elements (ie, blinding, study purpose, multicenter vs single-center), impact factor of the journal in which the study was published, and the interval from study completion to publication differed by study intervention type. These factors were selected as reflections of publication quality.^32^

### Sample Size

Sample size calculations were performed using the Russ Lenth Java applet for determining power and sample size.^33^ We assumed a worst-case scenario and estimated that 50% of registered probiotic trials would be published. Based on a survey of 9 colleagues (3 pediatric emergency medicine physicians; 3 pediatric infectious disease specialists; and 3 gastroenterologists), the median clinically meaningful difference in the proportion of studies published was set at 15%. Using a 2-sided α of .05, a minimum of 135 studies were required in each cohort to reject the null hypothesis with 80% power.

### Statistical Analysis

We report trial characteristics by exposure status (ie, probiotic or antibiotic) using descriptive statistics. Between-group comparisons for study design elements were performed using the Pearson χ^2^ test or the Fisher exact test if the number of studies in any single category was less than 10. For nonnormally distributed data, we used the Mann-Whitney test to compare 2 independent samples. Interrater reliability regarding publication status was determined using the Cohen κ value.^34^

For the primary outcome of publication status, we compared antibiotic and probiotic trials using the Pearson χ^2^ test. For the secondary outcomes, we conducted an a priori planned multiple logistic regression analysis to assess the association between publication status (dependent variable) and the type of intervention (ie, probiotic vs antibiotic) including the following a priori identified covariates: funding source, blinding, and study purpose. We compared features of study design, outcomes, and funding source between groups using the Pearson χ^2^ test. We used the Mann-Whitney test to compare impact factor of the journal in which the study was published and time to publication for the 2 groups.

Statistical analyses were completed on October 16, 2020, using SPSS Statistics Subscription Build, version 1.0.0.1461 (IBM Corp). Statistical significance was set at 2-sided *P* < .05.

## Results

**Figure 1.**
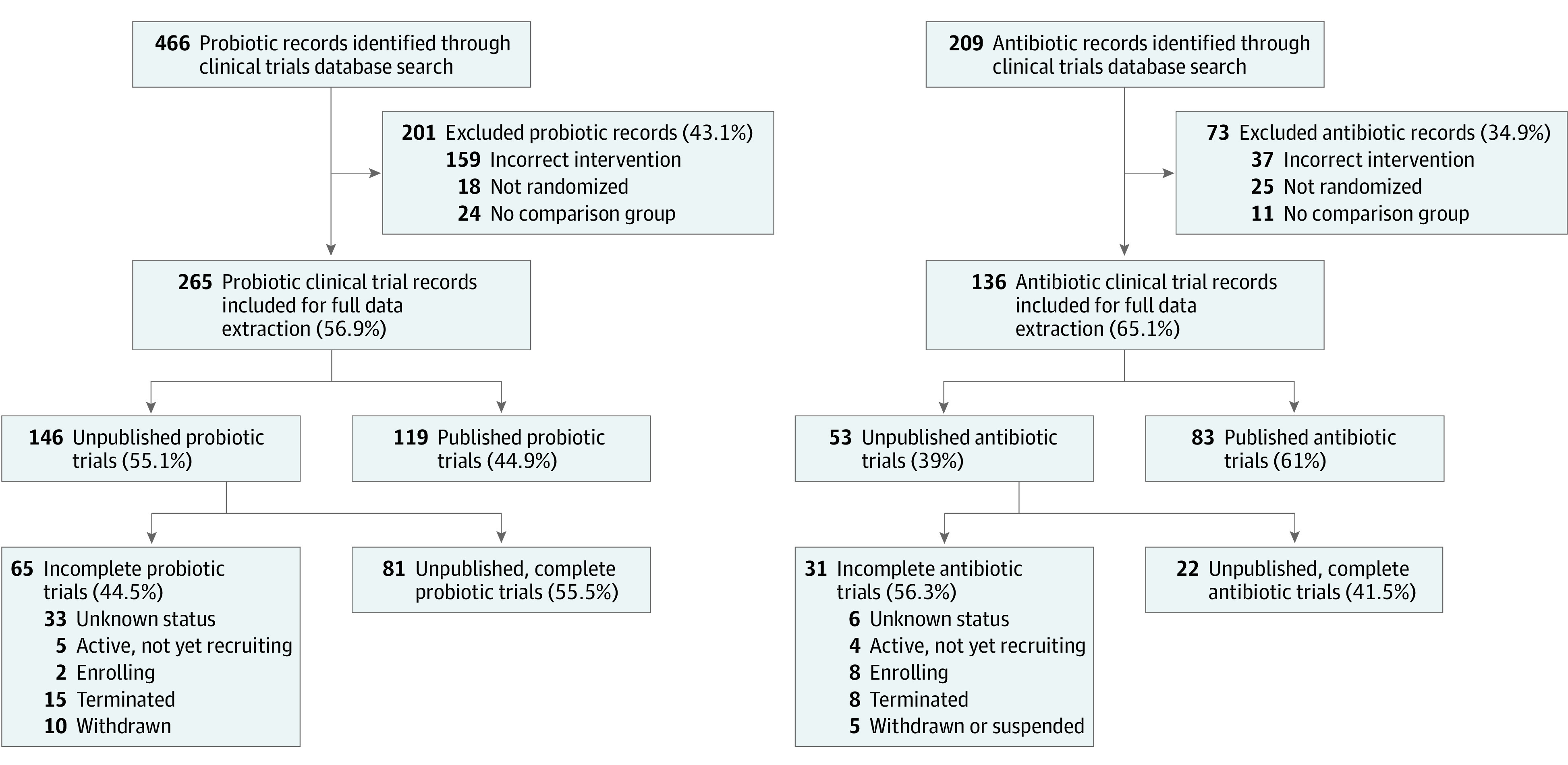
Flow Figure of Study Eligibility Assessment and Inclusion

We identified 466 unique probiotic and 209 unique antibiotic trials in ClinicalTrials.gov. After screening, 265 (56.9%) probiotic and 136 (65.1%) antibiotic records were retained for a total of 401 studies (Figure 1). Fifty-eight probiotic trials reported their status as withdrawn, terminated, or unknown compared with 18 antibiotic trials (21.9% vs 13.2%; difference, 8.7% [95% CI, 0.5%-15.8%]). Probiotic trials were of shorter duration (median, 537 [IQR, 306-973] days) compared with antibiotic trials (median, 1096 [IQR, 668-1642] days; *P* = .001). Probiotic trials were more likely to be placebo-controlled compared with antibiotic trials (205 [77.4%] vs 55 [40.4%]; difference, 36.9% [95% CI, 26.9%-46.1%]), whereas antibiotic trials were more likely to use a separate intervention (38 [27.9%] vs 18 [6.8%]; difference, 21.2% [95% CI, 13.4%-30.0%]) or dosing regimen (24 [17.6%] vs 3 [1.1%]; difference, 16.5% [95% CI, 10.6%-23.8%]) as comparison groups. Results were more commonly available in ClinicalTrials.gov for the antibiotic studies (39 [28.7%] vs 17 [6.4%]; difference, 22.3% [95% CI, 14.4%-30.7%]) (Table 1).

**Table 1.** Characteristics of Eligible Trials

Characteristic	Trial group^a^		*P* value
	Probiotics (n = 265)	Antibiotics (n = 136)	
Study purpose			
Therapeutic	117 (44.2)	114 (83.8)	<.001
Prophylactic	131 (49.4)	20 (14.7)	
Other^b^	17 (6.4)	2 (1.5)	
Blinding^c^	237 (89)^d^	96 (70.6)	<.001
Population			
Premature infants	54 (20.4)	4 (2.9)	<.001
Term infants	55 (20.8)	5 (3.7)	
All ages	123 (46.4)	75 (55.1)	
Children and adults	24 (9.1)	42 (30.9)	
Not specified	9 (3.4)	10 (7.4)	
No. of participants, median (IQR)^e^	110 (58-241)	205 (64-520)	.02
Study duration, median (IQR), d^f^	537 (306-973)	1096 (668-1642)	.001
Multicenter^g^	46 (17.4)	46 (33.8)	.001
Condition			
Gastrointestinal	69 (26.0)	NA	NA
Prematurity	59 (22.3)	NA	NA
Preventative	34 (12.8)	NA	NA
Allergy	26 (9.8)	NA	NA
Respiratory	NA	40 (29.4)	NA
Ears/nose/throat	NA	17 (12.5)	NA
Genitourinary	NA	16 (11.8)	NA
Gastrointestinal	NA	15 (11.0)	NA
Other	77 (29.1)^h^	48 (35.3)^i^	NA
Intervention			
Probiotic^j^			
* Lactobacillus reuteri*	46 (17.4)	NA	NA
* Lactobacillus rhamnosus*	27 (10.2)	NA	NA
* Saccharomyces boulardii*	5 (1.9)	NA	NA
*Bifidobacterium* species	12 (4.5)	NA	NA
Mixed	67 (25.3)	NA	NA
Other	71 (26.8)	NA	NA
Antibiotic			
Azithromycin	NA	70 (51.5)	NA
Amoxicillin	NA	32 (23.5)	NA
Amoxicillin/clavulanic acid	NA	17 (12.5)	NA
Cephalexin	NA	7 (5.1)	NA
Cefdinir	NA	3 (2.2)	NA
Mixed	NA	7 (5.1)	NA
Comparison group^k^			
Placebo	205 (77.4)	55 (40.4)	<.001
Standard care	39 (14.7)	19 (14.0)	
Separate intervention	18 (6.8)	38 (27.9)	
Alternate dosages	3 (1.1)	24 (17.6)	
Results provided in ClinicalTrials.gov	17 (6.4)	39 (28.7)	<.001
Industry funded	88 (33.2)	18 (13.2)	<.001

Abbreviation: NA, not applicable.

^a^ Unless otherwise indicated, data are expressed as number (%) of trials. Percentages have been rounded and may not total 100.

^b^ Includes basic science (9 probiotic and 1 antibiotic) and safety/tolerability (8 probiotic and 1 antibiotic).

^c^ Defined as any level of masking (single-blind or double-blind) reported in ClinicalTrials.gov.

^d^ One probiotic study did not state blinding.

^e^ Nine probiotic studies and 6 antibiotic studies had an actual enrollment of 0 and were therefore excluded from the analysis. In addition, 8 antibiotic studies were excluded owing to very large (>200 000) enrollment numbers.

^f^ Two hundred fifty-eight probiotic studies and 135 antibiotics studies included study start and end dates in ClinicalTrials.gov.

^g^ Two hundred thirty-two probiotics studies and 125 antibiotic studies disclosed location in ClinicalTrials.gov.

^h^ Includes congenital heart disease, cystic fibrosis, cerebral palsy, iron deficiency, peritoneal dialysis, pediatric burns, tuberculosis, dental, colic, obesity, surgical, vaccinations, and mental health.

^i^ Includes skin infections, postoperative infections, and sepsis.

^j^ Thirty-seven studies did not describe probiotic strain more specifically.

^k^ One probiotic trial used an antibiotic as the comparison group, and because this antibiotic was not one of the included antibiotic types for the current analysis, the study was included in the probiotic trial group.

### Primary Outcome

Interreviewer publication status classification agreement for antibiotic and probiotic studies was outstanding, with κ values of 0.94 (95% CI, 0.90-0.98) and 0.82 (95% CI, 0.75-0.89), respectively. A greater proportion of antibiotic studies were published compared with probiotic studies (83 [61.0%] vs 119 [44.9%]; difference, 16.1% [95% CI, 5.8%-25.9%]).

### Secondary Outcomes

After adjustment for industry funding, blinding, and study purpose, studies evaluating an antibiotic were more likely to be published (odds ratio, 2.1 [95% CI, 1.3-3.4]) (Table 2). Of the 83 published antibiotic studies, 56 (67.5%) reported statistically significant results, compared with 58 of the 119 published probiotic trials (48.7%; difference, 18.7% [95% CI, 4.9%-31.4%]). The proportion of industry-funded trials reporting statistically significant benefits was higher for antibiotic trials (10 of 13 [76.9%]) compared with probiotic trials (15 of 34 [44.1%]; difference, 32.8% [95% CI, 1.1%-54.1%]). A greater proportion (38 of 119 [31.9%]) of published probiotic trials were industry funded compared with published antibiotic trials (10 of 83 [12.0%]; difference, 19.9% [95% CI, 8.3%-30.2%]).

**Table 2.** Adjusted ORs for the Association Between Publication Status and Trial

Characteristic	No. (%) of trials		OR (95% CI)	*P* value
	Published (n = 202)	Unpublished (n = 199)		
Intervention				
Probiotic	119 (58.9)	146 (73.4)	2.1 (1.3-3.4)	.002
Antibiotic	83 (41.1)	53 (26.6)		
Industry funded	48 (23.8)	58 (29.2)	0.9 (0.5-1.4)	.54
Blinded^a^	166 (82.2)	167 (84.3)	1.03 (0.6-1.8)	.91
Study purpose^b^				
Therapeutic	116 (57.7)	115 (57.8)	0.9 (0.2-3.2)	.84
Prophylactic	76 (37.8)	75 (37.7)	1.3 (0.9-2.1)	.20
Other	9 (4.5)	9 (4.5)	2.8 (0.6-11.8)	.16

Abbreviation: OR, odds ratio.

^a^ One unpublished study did not specify blinding.

^b^ One published study did not specify study purpose.

**Figure 2.**
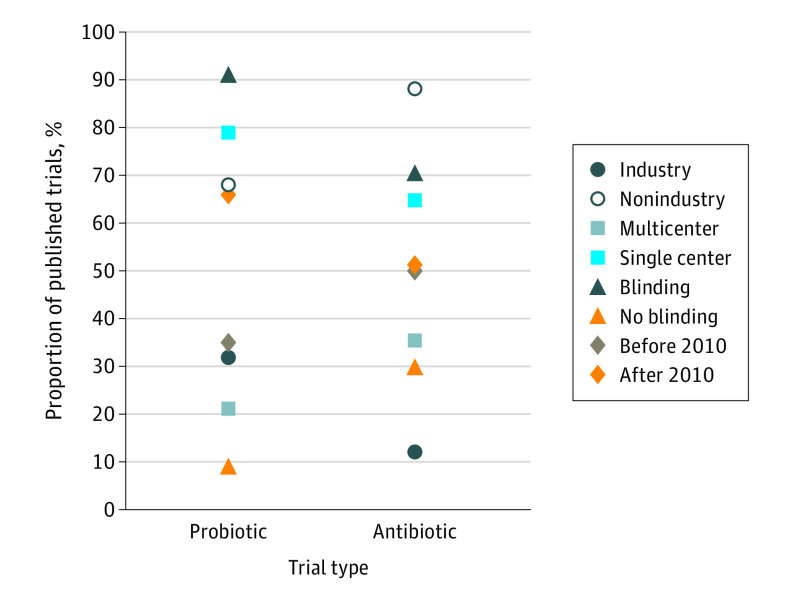
Publication of Antibiotic and Probiotic Studies Based on Funding Source, Blinding, and Multicenter Design

Antibiotic trials, compared with probiotic trials, were more likely to be therapeutic (114 of 136 [83.8%] vs 117 of 265 [44.2%]; difference, 39.7% [95% CI, 30.4%-47.5%]) and multicenter (46 of 136 [33.8%] vs 46 of 265 [17.4%]; difference, 16.5% [95% CI, 7.5%-25.7%]) (Table 1). A greater percentage of published antibiotic trials were multicenter in design (28 of 79 [35.4%]) compared with published probiotic trials (22 of 104 [21.2%]; difference, 14.2% [95% CI, 1.2%-27.2%]) (Figure 2). The median impact factor of the journals in which the articles were published was lower for the probiotic trials than antibiotic trials (3.0 [IQR, 2.3-4.2] vs 7.2 [IQR, 2.8-20.5]; *P* < .001) (eTable 4 in the Supplement). The median time to publication did not differ between the probiotic and antibiotic trials (801 [IQR, 550-1183] vs 683 [IQR, 441-1036] days; *P* = .24).

### Exploratory Analyses

Industry-funded trials were more likely to involve premature or term infants compared with non–industry-funded trials (45 of 106 [42.5%] vs 73 of 295 [24.7%]; difference, 17.7% [95% CI, 7.3%-28.3%]). Antibiotic trials with statistically significant results were more likely to be multicenter (24 of 58 [41.4%]) compared with statistically significant probiotic trial findings (7 of 48 [14.6%]; difference, 26.8% [95% CI, 9.6%-41.6%]). Although the proportion of antibiotic trials has not changed over time (through 2010, 67 of 136 [49.3%]; after 2010, 69 of 136 [50.7%]; difference, 1.2% [95% CI, −13.7% to 16.0%]), the proportion of registered probiotic trials that were published has increased (through 2010, 91 of 264 [34.5%]; after 2010, 173 of 265 [65.3%]; difference, 31.0% [95% CI, 18.5%-42.3%]).

## Discussion

In this study, we determined that antibiotic trials registered on ClinicalTrials.gov from July 1, 2005, to June 30, 2016, were more than twice as likely to be published compared with probiotic trials registered during the same period. Although characteristics such as funding source, study purpose, and blinding differed between probiotic and antibiotic studies, none of these were independently associated with publication status. The impact factor of the journal in which the studies were published was higher for the antibiotic studies.

Earlier evaluations of publication bias in the probiotic literature identified selective publication using statistical methods such as funnel plots and Egger intercept tests.^35,36^ One study^35^ reported that studies with negative findings were less likely to be published. The presence of significant publication bias^35,36^ led to a cautious interpretation of results that was reflected in the conclusions. We are unaware of any studies that have assessed publication bias in a field of research (eg, probiotics) by comparing publications with those of another field (eg, antibiotics). Although industry funding has been reported to influence the likelihood of publication,^37,38,39^ the only prior evaluation of this concern in the probiotic literature identified no such association.^40^ In our study, although industry funding was significantly greater among probiotic studies, it was not independently associated with publication status.

In general, pediatric studies are less likely to be published compared with studies that focus on adults.^39,41,42,43^ A study conducted in 2012^39^ that used a design similar to that of our study reported that only 29% of included pediatric trials resulted in publication. Furthermore, those authors found that industry-funded pediatric trials were less likely to be published compared with those funded by the National Institutes of Health.^39^ However, there does appear to be a trend toward increasing publication of pediatric studies. A review of abstracts accepted to the Pediatric Academic Societies’ annual meetings from 1992 to 1995 found that 41% of abstracts were unpublished as of the year 2000.^42^ When this analysis was repeated 15 years later, only 28% of studies presented at this same meeting from 2008 to 2011 remained unpublished as of 2017.^41^ Larger sample sizes and registration on ClinicalTrials.gov are 2 factors that may explain the higher publication proportions.

Although we determined that pediatric probiotic studies are less likely to be published compared with pediatric antibiotic studies, our results do not pinpoint the underlying explanation. Although others have demonstrated a bias toward the publication of beneficial results using the US Food and Drug Administration registry and results database,^44^ no such data are available for probiotic studies, which are rarely conducted with the intention of applying for marketing approval or a labeling change.^45^ Thus, it is possible that the unpublished probiotic studies represent negative trials and that the higher level of selective publication in this field renders the results of meta-analyses unreliable.^2^

Selective publication is possible in all fields of research, and this can lead to inaccurate estimates of the effectiveness of an intervention.^44^ Selective publication wastes limited resources and the contributions of study participants and hinders the advance of medical knowledge. By altering the assessment of efficacy, selective publication can lead to inappropriate medication use and recommendations. Attempts to study selective publication are hindered by the unavailability of data from unpublished trials.^44^ Although a variety of tests are available to detect selective reporting bias, none reliably detect or exclude the possibility of bias.^46,47,48,49,50^

### Strengths and Limitations

The strengths of our study include the large number of registered trials included for analysis that provide adequate power for our analyses. We designed our study to provide a large interval between study registration and assessment of publication status to provide sufficient time for studies to be completed and published. Our protocol for determining publication status and high interobserver reliability ensure confidence in labeling studies as unpublished. Our study is unique in that it compares selective publication across types of medications. This approach enables us to conclude that publication bias may be a greater concern in probiotic studies compared with those evaluating antibiotics in children.

A limitation of our study is the inability to determine the direction of results of trials that were unpublished and unavailable in ClinicalTrials.gov. Although the inclusion of conference abstracts in systematic reviews is a controversial topic,^51^ we did not include conference abstract publication in the definition of our primary outcome, which was focused on peer-reviewed journal publication indexed in PubMed or Google Scholar. We only used 1 comparison group—pediatric antibiotic trials—and ideally, subsequent analyses will include comparisons with other drug classes to enable similar comparative analyses to place this concern in the proper context. We also cannot conclusively determine the reason for nonpublication.

## Conclusions

The findings of this cross-sectional study suggest that registered pediatric probiotic trials are less likely to be published than registered pediatric antibiotic studies. Although further research is needed to establish the underlying study characteristics that explain this finding, our results raise a concern regarding the accuracy of meta-analyses that rely on published data to reach their conclusions. These results serve to highlight the importance of closing the loopholes and barriers that allow studies to be conducted without publishing their findings. This evidence should drive regulatory measures to ensure access to unpublished trial data and the provision of reasons for nonpublication.

## Notes

Supplement. **eTable 1.** Antibiotic Synonyms **eTable 2.** ClinicalTrials.gov Search Strategy Using Advanced Search Format **eTable 3.** Probiotic Synonyms **eTable 4.** Top 10 Journals of Publication Separated by Probiotic vs Antibiotic Trials
