# Supplementary material for: Comparison of Publication of Pediatric Probiotic vs Antibiotic Trials Registered on ClinicalTrials.gov
Source: JAMA Netw Open. 2021 Oct 8;4(10):e2125236. doi: 10.1001/jamanetworkopen.2021.25236 (PMC8501398; doi:10.1001/jamanetworkopen.2021.25236)
Supplement: Supplement. — eTable 1. Antibiotic Synonyms eTable 2. ClinicalTrials.gov Search Strategy Using Advanced Search Format eTable 3. Probiotic Synonyms eTable 4. Top 10 Journals of Publication Separated by Probiotic vs Antibiotic Trials [file jamanetwopen-e2125236-s001.pdf]

Supplementary Online Content

Riddell M, Lam K, Funk A, Lodha N, Lorenzetti DL, Freedman SB. Comparison of publication of pediatric probiotic vs antibiotic trials registered on ClinicalTrials.gov. *JAMA Netw Open*. 2021;4(10):e2125236. doi:10.1001/jamanetworkopen.2021.25236

eTable 1. Antibiotic Synonyms
eTable 2. ClinicalTrials.gov Search Strategy Using Advanced Search Format
eTable 3. Probiotic Synonyms
eTable 4. Top 10 Journals of Publication Separated by Probiotic vs Antibiotic Trials

This supplementary material has been provided by the authors to give readers additional information about their work.

© 2021 Riddell M et al. *JAMA Network Open*.

eTable 1. Antibiotic Synonyms

**Terms**	**Search Results[a]**	**Entire Database[b]**
Synonyms		
**Azithromycin**	99 studies	608 studies
Zithromax	33 studies	134 studies
Zmax	4 studies	19 studies
Azithromycine	3 studies	14 studies
AzaSite	2 studies	20 studies
Azitromicina	1 studies	1 studies
CP 62993	1 studies	3 studies
Aithromycin	--	1 studies
Azadose	--	1 studies
Azitrocin	--	1 studies
Sumamed	--	2 studies
Toraseptol	--	1 studies
Ultreon	--	1 studies
Vinzam	--	1 studies
Zentavion	--	1 studies
Zitromax	--	6 studies
**Amox-clav**	36 studies	197 studies
Amoxicillin Potassium Clavulanate Combination	29 studies	143 studies
Augmentin	18 studies	84 studies
amoxicillin clavulanate	12 studies	66 studies
co-amoxiclav	6 studies	22 studies
amoxycillin clavulanic acid	3 studies	31 studies
clavulin	3 studies	7 studies
Amoxicillin and clavulanate	1 studies	7 studies
Amoxi Clavulanate	--	1 studies
Clavamox	--	3 studies
**Amoxicillin**	97 studies	725 studies
Amoxil	2 studies	25 studies
Amoxicot	1 studies	1 studies
Clamoxyl	1 studies	5 studies
Dispermox	1 studies	1 studies
Moxatag	1 studies	1 studies
Moxilin	1 studies	1 studies
Trimox	1 studies	2 studies
Amoxicilina	--	1 studies
Amoxicillinum	--	1 studies
**Cefdinir**	6 studies	31 studies
Omnicef	4 studies	21 studies
**Cephalexin**	18 studies	74 studies
Keflex	7 studies	28 studies

© 2021 Riddell M et al. *JAMA Network Open*.

Biocef	1 studies	3 studies
zartan	1 studies	2 studies
Ed A-Ceph	--	1 studies
keftab	--	1 studies

[a] Number of studies in the search results containing the term or synonym
[b] Number of studies in the entire database containing the term or synonym

© 2021 Riddell M et al. *JAMA Network Open*.

eTable 2. ClinicalTrials.gov Search Strategy Using Advanced Search Format

**Probiotics**	**Antibiotics**
Other terms: Lactobacillus OR Probiotic OR Saccharomyces OR Enterococcus OR Streptococcus OR Acidophilus<br>Study Type: Interventional Studies (Clinical Trials)<br>Study Results: All studies<br>Recruitment: No limitations applied (included all not yet recruiting, recruiting, enrolling, active, not yet recruiting, suspended, terminated, completed, withdrawn, unknown status)<br>Age Group: Must include child (birth-17)<br>Sex: All<br>Locations: No restrictions applied<br>Additional Criteria: No restrictions applied (included all phases, all funder types, all study documents)<br>Results submitted: No restrictions applied (included all no results submitted, submitted, QC not concluded and submission with QC comments available)<br>Study Start: From 07/01/2005 to 06/30/2016	Other terms: Azithromycin OR Amox-clav OR Amoxicillin OR Cefdinir OR Cephalexin<br>Study Type: Interventional Studies (Clinical Trials)<br>Study Results: All studies<br>Recruitment: No limitations applied (included all not yet recruiting, recruiting, enrolling, active, not yet recruiting, suspended, terminated, completed, withdrawn, unknown status)<br>Age Group: Must include child (birth-17)<br>Sex: All<br>Locations: No restrictions applied<br>Additional Criteria: No restrictions applied (included all phases, all funder types, all study documents)<br>Results submitted: No restrictions applied (included all no results submitted, submitted, QC not concluded and submission with QC comments available)<br>Study Start: From 07/01/2005 to 06/30/2016

© 2021 Riddell M et al. *JAMA Network Open*.

eTable 3. Probiotic Synonyms

**Terms**	**Search Results**[a]	**Entire Database**[b]
Synonyms		
**Lactobacillus**	192 studies	1,100 studies
Acidophilus	21 studies	217 studies
lactic acid bacteria	4 studies	47 studies
lactobacilla	--	1 studies
**Probiotic**	274 studies	1,585 studies
**Saccharomyces**	11 studies	103 studies
Hemiascomycetes	--	1 studies
**Enterococcus**	20 studies	181 studies
enterococcal	3 studies	14 studies
**Streptococcus**	156 studies	722 studies
**Acidophilus**	192 studies	1,100 studies
Lactobacillus	185 studies	1,056 studies
lactic acid bacteria	4 studies	47 studies
lactobacilla	--	1 studies

[a] Number of studies in the search results containing the term or synonym
[b] Number of studies in the entire database containing the term or synonym

© 2021 Riddell M et al. *JAMA Network Open*.

eTable 4. Top 10 Journals of Publication Separated by Probiotic vs Antibiotic Trials

Journal Name (Impact Factor)	Number of probiotic trials published by journal	Number of antibiotic trials published by journal
New England Journal of Medicine (70.67)	2	12
Lancet (59.102)	0	3
JAMA (51.273)	0	3
BMJ (27.604)	1	1
Lancet Infectious Diseases (27.516)	0	1
Lancet Respiratory Medicine (22.992)	0	1
Gut (17.943)	0	1
Medicine (16.494)	1	0
Lancet Global Health (15.873)	0	3
Journal of Allergy and Clinical Immunology (14.11)	1	3
JAMA Pediatrics (12.004)	1	1

© 2021 Riddell M et al. *JAMA Network Open*.
